# Phylogeography of *Himalrandia lichiangensis* from the dry-hot valleys in Southwest China

**DOI:** 10.3389/fpls.2022.1002519

**Published:** 2022-10-17

**Authors:** Yaomei Qiao, Jian Liu, Xun Gong

**Affiliations:** ^1^ Key Laboratory for Plant Diversity and Biogeography of East Asia, Kunming Institute of Botany, Chinese Academy of Sciences, Kunming, China; ^2^ Key Laboratory of Economic Plants and Biotechnology, Kunming Institute of Botany, Chinese Academy of Sciences, Kunming, China; ^3^ University of Chinese Academy of Sciences, Beijing, China

**Keywords:** dry-hot valley, *Himalrandia lichiangensis*, Southwestern China, phylogeography, endemism

## Abstract

Both changing tectonics and climate may shape the phylogeographic patterns of plant species. The dry-hot valleys in southwestern China harbor a high number of endemic plants. In this study, we investigated the evolutionary history and potential distribution of an endemic shrub *Himalrandia lichiangensis* (Rubiaceae), to evaluate the effects of tectonic and climatic processes on this thermophilic plant species from the dry-hot valleys. By sequencing DNA from four plastid non-coding regions (*psb*M-*trn*D, *trn*D-*trn*T, *atp*B-*rbc*L and *acc*D-*psa*I) and the CAMX1F-CAMX2R region and ITS for 423 individuals from 23 populations, we investigated the genetic diversity, phylogeographical pattern and population dynamics of *H. lichiangensis*. We found a high degree of differentiation in *H. lichiangensis* during the middle Miocene (15-13 Myr), possibly triggered by the rapid tectonic uplift event in this period area. accompanied by frequent orogeneses in this period. This hypothesis is also supported by the association between genetic differentiation and altitudinal gradients among populations. The middle reach of the Jinsha River, which harbors the greatest genetic diversity, is most likely to have been a refugia for *H. lichiangensis* during Quaternary. We also detected a strong barrier effect between the Nanpan River and Jinsha River, suggesting the river system may play a role in geographical isolation between clades on both sides of the barrier. The Maximum Entropy Model (MaxEnt) results showed that future climate warming will lead to the niche expansion in some areas for *H. lichiangensis* but will also cause a scattered and fragmented distribution. Given the high among-population differentiation and no recent expansion detected in *H. lichiangensis*, its current phylogeographical pattern is possibly due to a long-term geographical barrier caused by uplifting mountains since the Miocene, as well as Quaternary climate refugia isolated also by high mountains. This study illustrated tectonic and climatic processes may have a continuous effect on plant phylogeography and offers insights into the origin of biodiversity and endemism in the dry-hot valleys of southwestern China.

## Introduction

The uplift of the Tibetan Plateau is one of the most significant geological events during the Cenozoic, altering both the Asian topography and climate (Guo et al., 2002; Su et al., 2014; Yuan et al., 2022). Since the Miocene, the southeastern Tibetan Plateau (STP) topography has been markedly changed (Zhang et al., 2016), forming many dry-hot river valleys that have influenced the distribution pattern of species (Yin et al., 2020). Because of the preponderant roles of geological and climatic events at different time scales, plenty of phylogeographical and biogeographic studies have emphasized these factors for understanding the biogeographic history, diversification, and intraspecific genetic structure of plant taxa (Hickerson et al., 2010). However, how these two processes act to influence the phylogenetic structure of some plant species remains to be studied (but see Kou et al., 2016).

The Hengduan Shan located in the STP, has long been considered a refuge for plants in the northern temperate zone during the Quaternary glacial periods, and it has become an important source and radiation area for plants (Wang, 2000). With the uplift of the STP, ridge-valley landform of this region has been shaped by several large rivers, and then leading to a unique vegetation adapted to this hot and dry climate (Harrison and Noss, 2017). The main rivers in the Hengduan Shan are the Salween (Nu River), Mekong (Lancang River), and Jinsha River and its tributaries (including the Yalong River, Dadu River, and Min River) in Yunnan and Sichuan Provinces (Sun et al., 2022). Despite the harsh dry climate of the dry-hot valleys in southwestern China, it harbors more than 1700 plant species, belonging to 752 genera and 165 families, most of them subtropical, with few tropical and warm-temperate species. In particular, the high endemism (ca. 37%) of plant taxa in the dry-hot valleys makes it a hypothetical refuge during the Quaternary glacial periods (Ou and Jin, 1996).

Understanding the origin, evolution and historical dynamics of endemic and endangered taxa from dry-hot valleys as well as the underlying mechanisms can help us to better unravel their evolutionary processes and manage their conservation. There are some studies regarding the genetic structure and speciation of xeric plants in the dry-hot valleys in southwestern China, such as shrub *Trailliaedoxa gracilis* (Rubiaceae) (Jia et al., 2016), small tree *Leucomeris decora* and *Nouelia insignis* (Asteraceae) (Zhao and Gong, 2015), shrub and *Hibiscus aridicola* (Malvaceae) (Zhang et al., 2019). However, these cases mainly focused on restricted regions and could not reveal the regionallly evolutionary relationship. One example is the modern disjunct distribution of *Terminalia franchetii* (Combretaceae) from the dry-hot valleys, which is associated with patterns of cpDNA haplotype variation, resulting from vicariance caused by several river separation and capture events (Zhang et al., 2011). Another study on *Sophora davidii* (Fabaceae) exemplified the utility of comparing chloroplast and nuclear DNA variability and suggested a major phylogeographical break in cpDNA associated with the “Tanaka-Kaiyong Line” (TKL), and that the complex geological and climatic changes in HDM area played a decisive role in the west-east differentiation of *S*. *davidii* (Fan et al., 2013). These previous studies suggested that geological events and biogeographical barriers had an indelible effect on genetic divergence and vicariant isolation of southwestern China, particularly in dry-hot valleys, while comprehensive studies are still limited in illuminating how both geological and climatic processes shaped the temporal phylogeographical patterns and evolutionary processes of the species assemblies of this region (Zhu et al., 2020).

**Figure 1:**
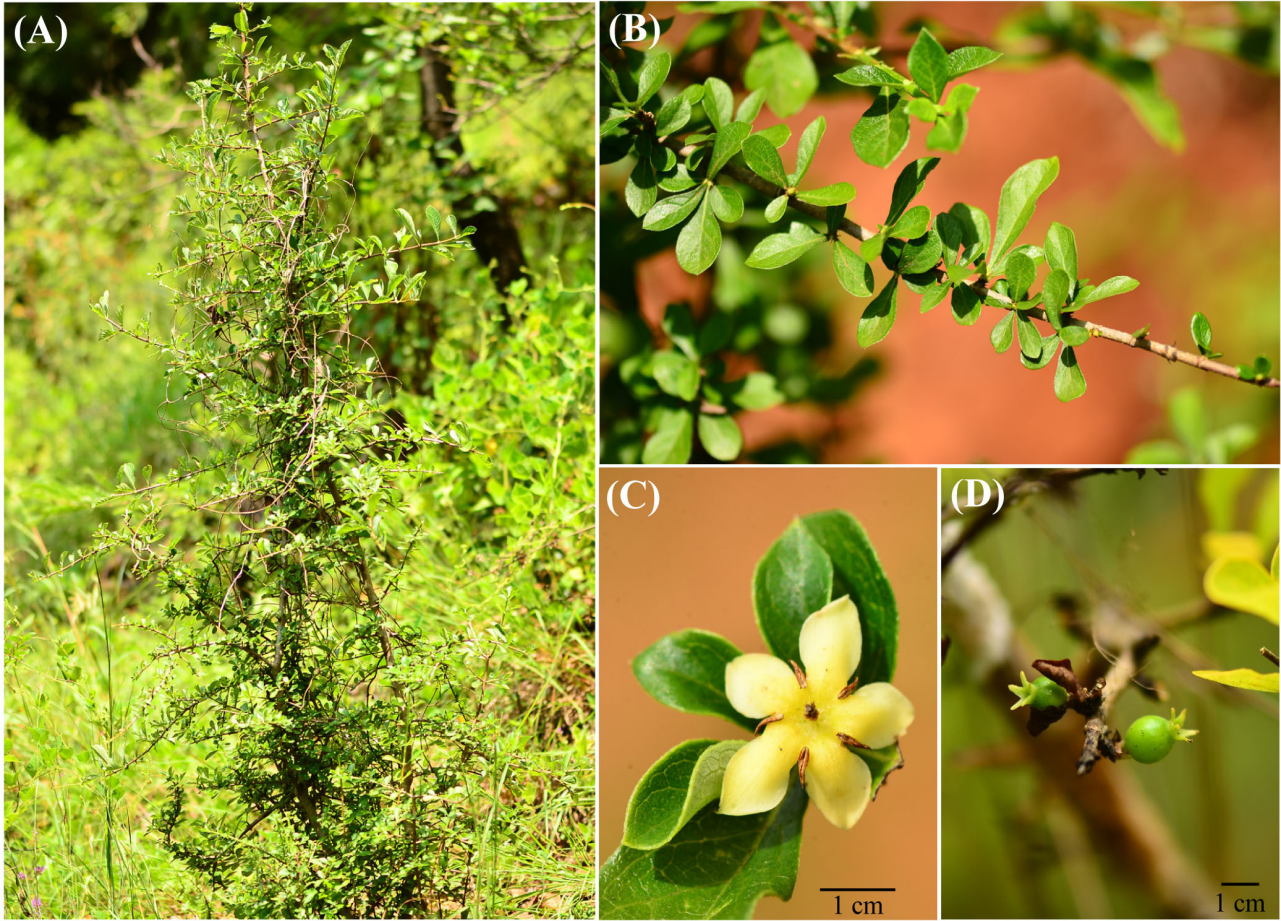
*Himalrandia lichiangensis* **(A)** Plant and habitat, **(B)** Leaves, **(C)** Flower, and **(D)** Fruit.

*Himalrandia lichiangensis* (W. W. Sm.) Tirveng. is an inermous perennial shrub (**Figure 1**) and is endemic to the dry-hot valleys in southwestern China, especially in the drainage area of the Jinsha River. Field survey indicated that its distribution range includes the drainage areas of the Jinsha River, Red River and Nanpan River, with an elevation range of 1200-2500 m. In this study, to investigate the phylogeography of this species, we established three major predictions: first, there should be significant genetic differentiation among the populations of *H. lichiangensis* owing to its low seed dispersal capability and the geographical isolation imposed by the mountains and rivers; second, considering its variable elevation and the preference for dry-hot valleys, we predicted both tectonic and climatic events impacted the phylogeographical pattern of *H. lichiangensis* in different periods; third, due to its thermophile nature, we predict the distribution of *H. lichiangensi*s may been affected drastically in the future because of global warming.

## Materials and methods

### Field sampling

We collected a total of 423 *H. lichiangensis* individuals from 23 sites covering almost the entire range of the species (**Table S1**). We chose 20 individuals from each population for genotyping, except for three populations, due to lack of individuals could not be found. Young and green leaf materials were stored with silica gel for DNA extraction. We designated two *Tarenna* species (*Tarenna attenuata* and *Tarenna depauperata*) from Rubiaceae as outgroup, which were sampled from cultivated materials kindly provided by the Xishuangbanna Tropical Botanical Garden, in Yunnan Province.

### DNA extraction, PCR amplification, and DNA sequencing

Genomic DNA was extracted from the silica-dried leaves using the CTAB method (Doyle, 1991). Four chloroplast DNA regions were amplified and sequenced: *trn*D-*psb*M, *trn*D-*trn*T, *acc*D-*psa*I, *atp*B-*rbc*L, using the primers described in a previous study (Shaw et al., 2007). Additionally, the coding region *CAMX*1F-*CAMX*2R (Strand et al., 1997) and the ITS4-ITS5 region (White et al., 1990) were amplified and sequenced for all samples. PCR and sequencing protocols were followed as in the studies in which those primers were described. A congruency test for the four combined cpDNA regions used in this study showed a significant rate of homogeneity (P > 0.5) by PAUP v 4.0b10 (Swofford, 2003), suggesting a high degree of homogeneity of the cpDNA regions by PAUP v 4.0b10. All cpDNA sequences were combined and regarded as a whole in the following analyses. Sequences were aligned using CLUSTAL X v.1.83 (Thompson et al., 1997) and double-checked manually. Nuclear genes often had heterozygous sites in some individuals, which were identified by overlapping peaks in chromatograms. The algorithms of PHASE (Stephens et al., 2001) in the software package DnaSP v.5.0 was used to resolve the nuclear sequences (Rozas et al., 2003).

### Genetic diversity, genetic differentiation and haplotype network construction

We calculated average within-population haplotype diversity (*H*_S_), total diversity (*H*_T_), the level of population differentiation (*G*_ST_ and *N*_ST_) as well as the genetic differentiation (*F*_ST_) at the species level. The U test was used to determine whether *N*_ST_ was significantly greater than *G*_ST_ (Pons and Petit, 1996). If *N*_ST_>> *G*_ST_, it indicated that there was significant phylogeographic structure among populations (Nei, 1973). All these parameters were calculated using the program Permut v.1.0 (Pons and Petit, 1996). The software GenALEx v.6.0 (Peakall and Smouse, 2006) was used to calculate the geographical distance between each population pair, and a Mantel test (Mantel, 1967) implemented in the GenALEx v.6.0 software was used to compare the correlation between geographical distance and genetic distance. We used Arlequin v.3.1 (Excoffier et al., 2005) to conduct the analysis of molecular variance (AMOVA) to estimate genetic variation within and among populations. To test whether *H. lichiangensis* has undergone recent demographic population expansion events, we calculated Tajima’s *D* (Tajima, 1996) and Fu’s *F*s (Yun, 1997) of each population and all populations together using the program DnaSP v.5.0 (Rozas et al., 2003). We also plotted the mismatch distribution as the observed number of differences between pairs of haplotypes using DnaSP v.5.0 (Rozas et al., 2003). These neutrality test statistics detect departures from population size equilibrium caused by population expansions or bottlenecks (Korneliussen et al., 2013).

Genealogical relationships among haplotypes were inferred from an unrooted statistical parsimony haplotype network as estimated by NETWORK v.4.2 (Bandelt et al., 1999), with 95% most parsimonious connection. The geographical distribution of haplotypes and sampling localities were plotted in ArcGis v.10.5 (https://developers.arcgis.com). Phylogenetic reconstructions of ITS ribotypes were implemented in IQTREE v.2.1.1 (Minh et al., 2020) to infer the maximum-likelihood (ML) tree using the ultrafast bootstrap approximation method (Hoang et al., 2018) with the best fitting GTR + F + I model and 1000 bootstrap replicates. The abundance of ITS ribotypes across the 23 populations was depicted using the online program iTOL (https://itol.embl.de; Letunic and Bork, 2019). In addition, we tested whether significant difference in altitude exists between population groups revealed by ITS haplotypes. A simple t-test was performed among groups by using R package *ggsignif* (Lan et al., 2019).

### Divergence time estimation and BARRIER analysis

Before the haplotype divergence time inference, we first investigated the systematic position of *H. lichiangensis* in Rubiaceae by phylogenetic reconstruction using *rbc*L gene (Bremer et al., 1995). *rbc*L gene data of 40 species from three subfamilies of Rubiaceae were downloaded from NCBI, and a Bayesian Inference (BI) was conducted with two species as outgroup (*Ligustrum vulgare* and *Kopsia fruticosa*). BI analysis was conducted in MrBayes 3.2 (Ronquist et al., 2012) by running 10^6^ generations with every 100th generation sampled from the chain. The first 20% of the trees were discarded as burn-in, and the remaining were used to generate a majority-rule consensus tree and estimate the posterior probability. The program jModeltest v.2.1 (Darriba et al., 2012) was used to determine the best-fitting model for the matrix of sequences. The Bayesian tree of Rubiaceae using the *rbc*L gene showed a close relationship of *H. lichiangensis* and *T. drummondii* (in the same genus as the outgroup species *T. depauperata* and *T. attenuate* used in this study, **Figure S1**). Age estimation was implemented in BEAST v.1.8.4 (Drummond and Rambaut, 2007). We chose the uncorrelated lognormal relaxed clock model as the clock model because it was favored by the prior nested sampling (NS) model selection test in BEAST. BEAST was run for a total of one billion generations with a sampling frequency of 5,000 generations. Tracer v.1.6 was used to check the convergence of chains to the stationary distribution (effective sample size > 200). The first 2,500 trees were discarded as burn-in, the remaining trees were summarized as a maximum clade credibility tree using TreeAnnotator. Using relaxed molecular clock, we further calibrated the common ancestors of *H. lichiangensis* and *Tarenna drummondii* as diverging at about 27.3 Ma (95% highest posterior density, HPD:21.3-33.3 Ma) according to a previous molecular dating study (Bremer and Eriksson, 2009). To test if physical barriers (river system) have impending influence to gene flow, we used BARRIER v.2.2 (Manni et al., 2004) to detect whether significant geographic isolation barriers exist between different populations. We used Arlequin v.3.1 (Excoffier et al., 2005) to calculate the matrix of Nei’s genetic distance (Nei et al., 1983) between populations, and 1000 repetitions were run for the significance test of each potential barrier.

### Ecological niche modelling

To reconstruct past and future shifts in the distribution of *H. lichiangensis*, we estimated its potential distribution range in four periods (Last Inter Glacial, LIG; Last Glacial Maximum, LGM; present; future, 2070s) using all available 23 presence records by niche modeling based on 19 bioclimatic variables (Zhang et al., 2020) (http://www.worldclim.org/) and altitude data (http://www.geodata.cn). The maximum spatial resolution of 19 bioclimatic and altitude data is 30"×30" (~ 1 km^2^), and the LGM and future data are derived from MIROC climate model (Watanabe et al., 2011), LIG data from Community Climate System Model (CCSM). For climate data in the 2050-2070 period, we selected the carbon dioxide concentration pathway RCP8.5 (Ford et al., 2012). The maximum entropy method (MaxEnt; Elith et al., 2006) was used to build a niche model for *H. lichiangensis* (Hernandez et al., 2006). Models were estimated from the average of 10 replicates and model performance was estimated using 25% of the points to test the model; the remaining points were used for training. Model performance was evaluated using the area under the curve (AUC) of the receiver operating characteristics (ROC) curve following Ortega-Huerta and Peterson (2008). To minimize biased fitting of the niche models, pairwise correlations among the 20 variables were calculated using the SPSS software (IBM, Shanghai, China). Only variables with a correlation coefficient r < 0.7 were selected. For variable pairs with r > 0.7, one of the two variables was selected (Yang et al., 2022). Finally, seven environmental variables (bio2, bio4, bio6, bio12, bio13, bio14 and altitude data) were selected to construct the models. ArcGIS v.10. 5 software was used to visualize the Maxent model operation result graph, and divide the suitability distribution area. Suitability was divided into three grades by reclassify tools in ArcGIS v.10. 5 (Yang et al., 2013): lowly suitable (0-0.4); sub-suitable (0.4-0.6); highly suitable (0.6 ~ 1).

## Results

### Sequence characteristics, genetic diversity and differentiation

For all individuals, the aligned sequences lengths of the combined cpDNA, ITS and *CAMX* regions were 3448 bp, 643bp and 854bp, respectively, and 19 cpDNA, 21 ITS and 15 *CAMX* haplotypes were identified, respectively. Genetic diversity indices of total nucleotide (*P*i) and haplotype diversity (*H*d) for all populations were summarized in **Table S1**. Total genetic diversity (*H*_T_ = 0.952, 0.785, 0.812 from cpDNA, ITS and *CAMX*, respectively) was higher than the average intrapopulation diversity (*H*_S_ = 0.124, 0.197 and 0.053 from cpDNA, ITS and *CAMX*, respectively), resulting in high levels of genetic differentiation (**Table 1**). The U tests showed that *N*_ST_ was significantly greater than *G*_ST_ (P = 0.006, 0.005, 0.044 for cpDNA, ITS and *CAMX*, respectively), implying that there is correspondence between haplotype lineages and geographic distribution. The results of the Mantel test revealed the same pattern, namely, there is a significant positive correlation between genetic and geographical distance (cpDNA: r = 0.182, P < 0.05; ITS: r = 0.354, P < 0.05; *CAMX*: r = 0.331, P < 0.05), which indicated that the genetic pattern of this species was in accord with the Isolation by Distance model (IBD; **Figure S2A**).

**Table 1:** Genetic diversity, differentiation indices for the combined cpDNA and nDNA in this study.

Markers	*H*_S_	*H*_T_	*G*_ST_	*N*_ST_
cpDNA	0.124 (0.0410)	0.952 (0.0225)	0.925 (0.0317)	0.959 (0.0228)
ITS	0.197 (0.0592)	0.785 (0.0493)	0.749 (0.0696)	0.842 (0.0562)
*CAMX*	0.053 (0.0337)	0.812 (0.0607)	0.934 (0.0398)	0.960 (0.0276)

*H*_T_, total genetic diversity; *H*_S_, average intra-population diversity; *N*_ST_ and *G*_ST_, gene differentiation coefficient.

The AMOVA revealed that 89.80% of the genetic variation was presented among populations and 10.20% was within populations at the cpDNA level, consistent with the nDNA data, meaning that genetic variation existed predominantly among populations (**Table 2**). In addition, we detected high level of genetic differentiation (*F*_ST_ = 0.898, 0.846, 0.957 from cpDNA, ITS and *CAMX*, respectively). The results of the mismatch distribution analysis displayed a multimodal distribution pattern (**Figure S2B**) with non-significant positive values of SSD (**Table 3**), which indicates that the species has not undergone a recent population expansion. This conclusion is also supported by neutrality test, which yielded positive values in most results (**Table 3**).

**Table 2:** Analysis of molecular variance (AMOVA) based on cpDNA and nDNA haplotype frequencies for populations.

Markers	Source of variation	df	Sum of squares	Variance components	Percentage of variation	*F*_ST_	*N*m
cpDNA	Among populations	22	991.146	2.32600 Va	89.80%	0.898	0.029
	Within populations	400	110.750	0.26432 Vb	10.20%		
ITS	Among populations	22	1353.471	1.66622 Va	84.55%	0.846	0.046
	Within populations	823	326.900	0.39721 Vb	15.45%		
*CAMX*	Among populations	22	1035.059	1.22052 Va	95.68%	0.957	0.011
	Within populations	823	47.550	0.05516 Vb	4.32%		

*F*_ST_, genetic differentiation; *N*m, gene flow; df, degree of random.

**Table 3:** Parameters of neutrality tests and mismatch analysis (*P<0.05).

Markers	Tajima’s D	Fu’s *Fs*	Raggedness	SSD
cpDNA	2.00446	3.232	0.0177	0.04772
ITS	2.61304*	-0.015	0.1357	0.01788
*CAMX*	0.82801	0.148	0.0391	0.00202

SSD, The variance of an observation of a point of difference with an expected value.

### Haplotype distribution patterns and phylogenies

**Figure 2:**
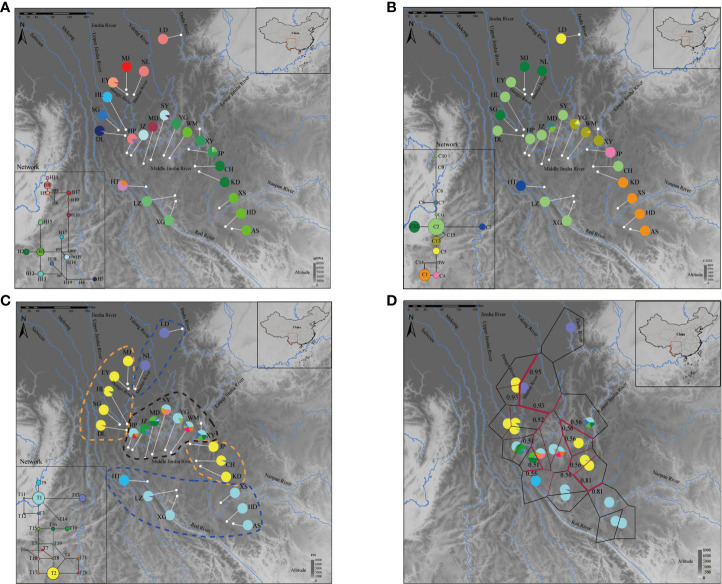
Geographical distribution of haplotypes and BARRIER analysis of *H. lichiangensis*. **(A–C)** Haplotype distributions for 23 populations based on **(A)** cpDNA, **(B)** *CAMX*, and **(C)** ITS data matrices. Frequencies of haplotypes in each population are indicated by the pie diagrams. The lower left inset of each panel are results from haplotype network analyses. **(D)** BARRIER analysis based on combined nDNA data. The detected barriers are indicated by heavy red lines. Numbers near the lines indicate bootstrap support.

For cpDNA (**Figure 2A**), H1 was the most frequent haplotype, distributed among all populations and showing a conspicuous disjunct distribution pattern between the Jinsha River and southern Nanpan River systems. In contrast, H8 was mainly found in Middle Jinsha River (NL, HP), Dadu River (LD) and the Red River (HT). It is noteworthy that LD is the only population found in the Dadu River basin, which is the tributary of Lower Jinsha River. In addition, H13 is found only in the Middle Jinsha River (YG, JP and XY), and so is H14. For the nuclear region *CAMX*, 15 haplotypes were detected (**Figure 2B**). C2 was the most dominant haplotype (Jinsha River: CH, DL, EY, HL, HP, JP and SY; Red River: LZ, XG). Of the 15 haplotypes, 10 were private haplotypes (C3, C4, C6-C11, C14 and C15), which mainly occurred in the Middle Jinsha River (JP, MD, WM and YG) and the Red River (HT). For the ITS, a total of 21 haplotypes were detected (**Figure 2C**). T1 is shared by three basins, including the Red River (LZ, XG), Nanpan River (XS, AS and HD) and the Middle Jinsha River (HP, MD, SY, WM, XYand YG). Furthermore, a total of 11 private haplotypes (T4, T7, T9-T12, T14, T17, T18, T20 and T21) were found, mainly distributed in the Middle Jinsha River (DL, HP, JZ, SY and WM) and the Red River (HT, LZ).

**Figure 3:**
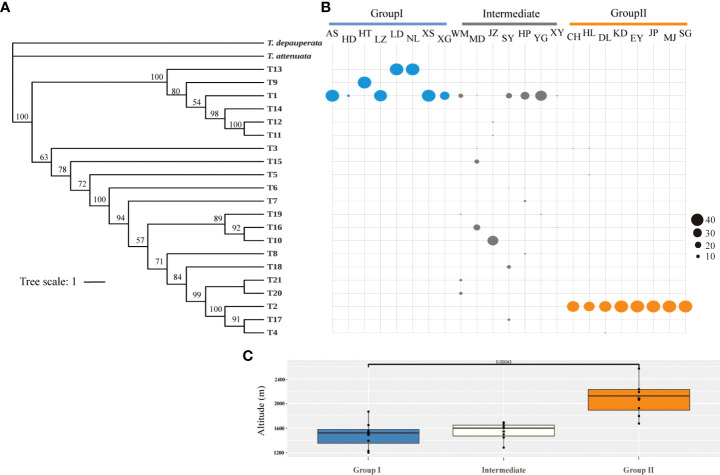
The abundance of ITS haplotypes and altitudinal variation between different population groups. **(A)** tree on the left represents the phylogenetic relationships among haplotypes. **(B)** ITS haplotypes abundance across 23 populations. Horizontal lines correspond to the haplotypes shown on the phylogenetic tree. Haplotype abundance in each population (vertical lines) is indicated by diameter of the circles, following the scale shown at the right. **(C)** Boxplot of altitude for the three groups of *H. lichiangensis*. Boxplot of altitude for the three groups of *H. lichiangensis*. Box-and-whisker plots indicate the median (horizontal line), 25th and 75th percentiles (bottom and top of the box), and limits of the 95% confidence intervals (lower and upper whiskers). Dots beyond the 95% confidence intervals are outliers. *P < 0.05.

Two groups and one intermediate group were determined by ITS haplotypes according to the distribution patterns (Group I: AS, HD, HT, LZ, LD, NL, XS and XG; Group Π: CH, HL, DL, KD, EY, JP, MJ and SG; intermediate: WM, MD, JZ, SY, HP, YG and XY) (**Figure 2C**). Moreover, the result of haplotypes abundance revealed that haplotype diversity was lower in both Group I and Π, while the haplotype diversity was higher in the intermediate group (**Figure 3B**). It is not difficult to find that the altitude differences among the three groups were significant according to the t-test results (**Figure 3C**). The phylogeny produced from the analysis of 21 ITS haplotypes was well-supported (**Figure 3A**). It was shown that T13, T9 and T1 were the earliest diverging haplotypes, and these haplotypes were distributed at lower elevations.

Network analysis of the cpDNA haplotypes suggested H1 is the haplotype with the most frequent occurrence and is located at the center of the reticular network tree. Based on the network analysis of the *CAMX* haplotypes (**Figure 2B**), the haplotype C2 that is located at the center appears to be the most frequent one. The HT population from the Red River basin has a private haplotype C3. The network analysis of ITS indicates T1 and T2 are the two most frequent haplotypes (**Figure 2C**).

### BARRIER and divergence time estimation

**Figure 4:**
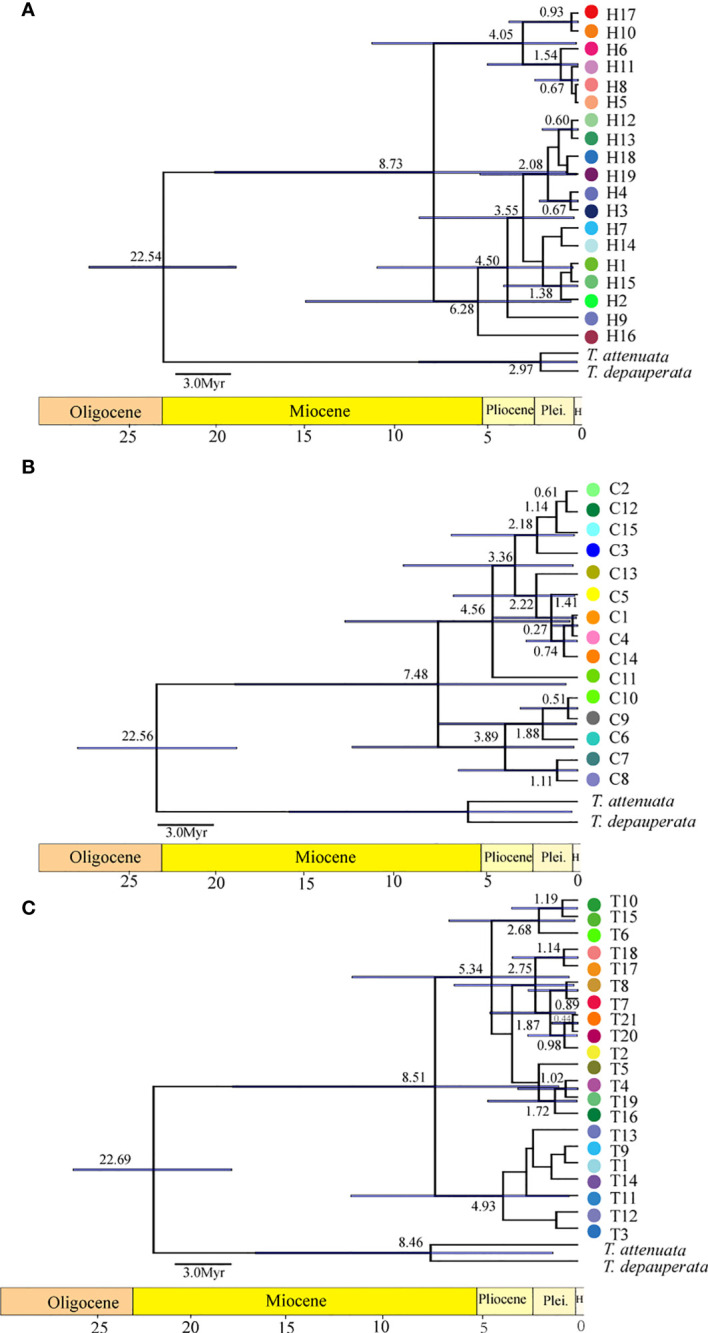
The chronogram of *H. lichiangensis* haplotypes reconstructed by BEAST. The BEAST trees are inferred by using a second calibration based on **(A)** cpDNA, **(B)** *CAMX*, **(C)** ITS data matrices. Tip labels refer to the haplotype number as shown in **Figure 1** **(A–C)**. The bar on the node depicts the 95% HPD (highest posterior density interval) in age estimation. The bar under each chronogram depicts the geological timescale. Pleis, Pleistocene; H, Holocene.

The results of BARRIER analysis show that there are two obvious isolation barriers with high bootstrap values (**Figure 2D**): one is roughly located between Dongyi River populations (MJ and EY) and Shuiluo River (NL), and the other barrier separates the Nanpan River populations (AS, XS) and Jinsha River populations (KD, CH). BEAST results based on both cpDNA and nDNA datasets (**Figure 4**) all showed that the first divergence of *H. lichiangensis* occurred during the middle Miocene (15-13Myr). The divergence of most haplotypes were recent with an estimate generally in the Pleistocene (around or later than 2 Myr).

### Ecological niche modelling

**Figure 5:**
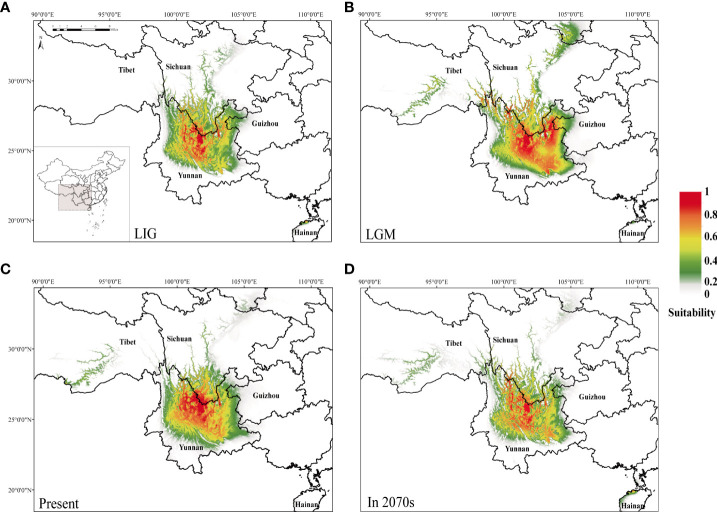
Potential distribution of *H. lichiangensis* in different periods. The prediction was conducted using Ecological Niche Modelling in four periods: **(A)** LIG: Last Inter Glacial, **(B)** LGM, Last Glacial Maximum, **(C)** present day, and **(D)** 2070s.

The accuracy of MaxEnt model was tested by receiver operator characteristic (ROC), and the areas under receiver operator characteristic curves (AUC) value was greater than 0.980 (**Figure S3**), indicating that the model had high stability and accuracy under the condition of a sample size of 23. The simulation results show that the current distribution area is well captured by the niche model (**Figure 5**). Under the climatic condition of the LIG, the optimal distribution area was greatly reduced compared with the current distribution area (**Table 4**). During the LGM, the suitable area expanded significantly (**Table 4**), reaching the farthest north to Gansu (**Figure 5B**). Surprisingly, under the future climatic conditions (in 2070s), the suitable are *lichiangensis* may shrink, accompanying by a large degree of habitat fragmentation (**Figure 5D**).

**Table 4:** Prediction of potential suitable distribution areas of *H. lichiangensis* in four periods.

Period	Prediction area (km^2^)			
	Lowly suitable area	Sub-suitable area	Highly suitable area	Total suitable area
LIG	115189.49	96173.57	39071.18	250434.24
LGM	129848.64	116751.28	58764.01	305363.93
Present	9985130.44	200021.23	51352.71	10236504.38
2070s	97782.97	57273.82	42223.53	197280.32

LIG, Last Inter Glacial; LGM, Last Glacial Maximum.

## Discussion

### Low genetic diversity and high differentiation by fragmentation and isolation

The fragmentation of habitats leads to the separation of ancestral populations, and the effect of genetic drift in a small population is likely to be more significant (Tsaliki and Diekmann, 2010). For *H. lichiangensis* that has a high fragmented distribution range and small population sizes, our results revealed that the genetic diversity within some populations (such as AS, HD, XS from the Nanpan River basin) is extremely low. The low genetic diversity of these populations could be explained by genetic drift, in which few haplotypes were fixed because of a bottleneck effect (Escudero et al., 2019). Generally, species in fragmented habitats tend to have lower genetic diversity within populations and high genetic differentiation among populations, which is in accordance with our results. In addition, of the 23 populations studied, nine contained only one haplotype (AS, HD, KD, LD, MJ, NL, SG, XG, and XS) for both cpDNA and nDNA, which further explained the bottleneck effect or genetic drift caused by small populations (Lefevre et al., 2004).

Geographical isolation, mutation, turbulent environmental factors and isolation of gene flow in plant populations can give rise to differences in the genetic structure of populations, and eventually lead to population differentiation (Jacquemyn et al., 2010). In this study, we found very high genetic differentiation among populations of *H. lichiangensis* with Fst value above 0.8, a degree that may give rise to speciation (Turner et al., 2005). However, we failed to distinguish morphological differences in *H. lichiangensis* specimens from highly diverged clades/populations. Despite this, we cannot exclude the possibility that divergence may have occurred in micromorphology or unexamined traits traits, and a more detailed and carefully-designed species delimitation study should be carried out in the future given the high genetic divergence of populations suggested by this work.

### Early divergence associated with geological events

The topography and climate changes have long been considered as the most fundamental drivers of plant diversity (Severin et al., 2015). Combining the haplotypes abundance analysis and t-test results in this study (**Figure 3**), it is not difficult to find that the genetic structure is to some extent affected by altitudinal gradient, namely, the heterogeneous uplift of HDM has driven differentiation of populations at contrasting elevations. Further significant increases in altitude of the Tibetan plateau are thought to have occurred about 10-8 Myr ago or more recently (An et al., 2001).We speculated that *H. lichiangensis* may have been distributed continuously and widely in the STP until the beginning of the Miocene (~23Myr). The rapid uplift of the STP since the middle Miocene blocked the gene flow among populations, and resulted in isolation, independent evolution and development of lineages in fragmented distribution regions (Harrison and Noss, 2017). Based on the results of BEAST, we estimated that the time of lineage differentiation was about 15-13 Myr (**Figure 4**), coinciding with the time of rapid uplift of QTP (An et al., 2001; Wang et al., 2021). Similar patterns have been found in other plant groups in the STP and adjacent region. For instance, the barrier of high mountain valleys formed by the uplift of QTP led to the differentiation of the two lineages in *Sinopodophyllum hexandrum* (Li et al., 2011). Similarly, a significant isolation-by-distance effect was detected across the entire distribution of *Prunus mira* populations, but geographic altitude might have more significant effects on genetic structure than geographic distance in partial small-scale areas (Bao et al., 2017). Besides, the genetic distribution pattern of *Taxus wallichiana* suggested that the barrier of the Hengduan and Dabashan mountains may have caused allopatric speciation (Gao et al., 2007). Therefore, early genetic divergence of *H. lichiangensis* was most likely attributed to the heterogeneous uplift in the STP, and the subsequent microevolutionary processes at the population level may be driven by effects of heterogeneous habitats such as topography, altitude and humidity (Alencar and Quental, 2021).

### Glacial refuge prediction and recent haplotype diversification of *H. lichiangensis*

Regions with high genetic diversity may indicate long-term survival of populations that have had a longer evolutionary history than populations that expanded after the glaciations, which is often thought of as a possible refuge for plants (Stephen et al., 2014). Previous studies have suggested that some species with a refuge located on the eastern edge of the Tibetan Plateau may have undergone expansion process after LGM (Last Glacial Maximum). A typical example is *Pedicularis longiflora*, which expanded its range from the eastern fringe (the refuge) to the plateau, accompanied by a subsequent continuous founder effect that significantly reduced the population diversity on the plateau, especially in the west (Yang et al., 2008). Similarly, the Qilian Mountain area was inferred as a potential glacial refuge, and the southern Tibet valley was considered as a ‘microrefugia’ for *Iris loczyi* (Zhang et al., 2021). According to the phylogeographical pattern of *H. lichiangensis*, we found both nDNA and cpDNA had high genetic diversity and more endemic haplotypes, however, in the middle reaches of Jinsha River, making this area likely to be a glacial refuge. These findings provided insights into the location of glacial refuges for the species distributed in STP and supplemented more plant species data for the response of species to the Quaternary climate (Sakaguchi et al., 2021).

The divergence of different haplotypes of *H. lichiangensis* mostly occurred during Quaternary (**Figure 4**), corresponding to the glacial refuge period discussed above. Combined with the strong barrier effect between the Nanpan River and Jinsha River as detected by the BARRIER analysis (**Figure 2D**), we conclude that the Quaternary climate change may have drastically impacted the recent genetic divergence of *H. lichiangensis*, suggesting the river system played an important role in geographical isolation and persisting the clades (haplotypes of populations) to both sides of the barrier during glaciation. Besides, populations located in marginal distribution zones (such as AS, HD, XS in the Nanpan River basin) have significantly reduced genetic diversity, most likely as a result of refuge population migration.

### Ecological niche modelling

The predicted potential current distribution range of *H. lichiangensis* is larger than the actual distribution. For instance, the Yarlung Zangbo River and The Mekong River also show the existence of suitable distribution area. This may suggest the limited distribution of *H. lichiangensis* is not restricted by present climate condition, but may be caused by habitat loss according to our field survey. During the Quaternary glaciations, high latitude species were affected by the spread of large ice sheets, which fragmented the geographical distribution of many species (Wang et al., 2013). While for some regions with relatively low latitude in China, the complex terrain and mountain barriers prevented the south from being covered by large ice sheets, making it one of the most important refuges for the southward retreat for many Tertiary relics (e.g., some gymnosperms, Kusky et al., 2011) in East Asia during the Quaternary glacial period. Based on the prediction of the suitable distribution area for *H. lichiangensis* in each period, LGM has the largest potential area (**Figure 5** and **Table 4**). Hence it seems low temperature in the glacial period does not cause the contraction of distribution areas, but provides conditions for expansion and possible migration for this thermophilic species.

Besides, the suitable distribution area shared by the species at different periods tend to be refuge for the species (Tang et al., 2018). Our Ecological Niche Modelling prediction demonstrates that the major distribution area of *H. lichiangensis* is relatively stable across all different periods, with a major potential occurrence in the midstream of Jinsha River and the upper reaches of the Red River (**Figures 5A–D**). However, contradicted to our prediction, the potential distribution of *H. lichiangensis* will become more scattered and fragmented than present in the face of future global warming, despite few expansions were detected (e.g., to Hainan Island, **Figure 5**). The contraction pattern of *H. lichiangensis* is consistent with the modelling result of another endangered species *Pseudotaxus chienii* from the south and southwestern China (Zhang et al., 2020). These results, taken together, imply that the endangered species with limited distribution range and distinct niche preference will be more sensitive to future climate change and prone to suffer more severe fragmentation, even for the thermophilic species like *H. lichiangensis*. Thus, immediate conservation biology studies on pollination ecology and reproductive biology should be undertaken, and practical conservation strategies are needed to avoid the shrink or even extinction of *H. lichiangensis* in the future.

## Conclusion

In this study, we found high genetic differentiation of *H. lichiangensis* among populations, which is possibly caused by long-term geographical isolation as no recent expansion was detected. It is worth noting that the current population genetic structure of *H. lichiangensis* is closely related to the elevation gradient, reflecting the influence of orogeny of STP in Miocene driven by complex tectonic movements. The Quaternary climate change that isolated refuges, together with several river barriers (such as Jinsha and Nanpan rivers), may have accelerated the diversification of different haplotypes. The largest past distribution of *H. lichiangensis* in LGM implied a trend of range expansion during the ice age and contraction after the glacial epoch. Climate warming in the coming 50 years will lead to a large decrease in the area suitable for *H. lichiangensis*, which offers imperative thinking for niche conservation and ecological restoration in dry-hot valley areas of southwestern China. The influence of geological and climatic events on the plant distribution in the dry-hot valley area needs to be extended to more plant groups with long evolutionary history in order to better understand how different factors may interact to impact the evolutionary process.

## Data availability statement

The original contributions presented in the study are publicly available. This data can be found here: GenBank, MW222416-MW222467, MW264453-MW264480.

## Author contributions

XG and JL conceived the study. YQ, JL, and XG participated in sampling. YQ performed the analyses and drafted the initial article, which was then corrected and finalized by JL and XG. All authors contributed to the article and approved the submitted version.

## Funding

This work was funded by the National Key Research and Development Programme of China (2017YF0505200), the National Natural Science Foundation of China (31900184 and 31970230), and the Natural Science Foundation of Yunnan Province (202001AT070072 & 2019FD057).

## Acknowledgments

We thank Zichen Zhao and Jie Song for their sampling assistance.

## Conflict of interest

The authors declare that the research was conducted in the absence of any commercial or financial relationships that could be construed as a potential conflict of interest.

## Publisher’s note

All claims expressed in this article are solely those of the authors and do not necessarily represent those of their affiliated organizations, or those of the publisher, the editors and the reviewers. Any product that may be evaluated in this article, or claim that may be made by its manufacturer, is not guaranteed or endorsed by the publisher.
