# Supplementary material for: Phylogeography of Himalrandia lichiangensis from the dry-hot valleys in Southwest China
Source: Front Plant Sci. 2022 Oct 17;13:1002519. doi: 10.3389/fpls.2022.1002519 (PMC9618719; doi:10.3389/fpls.2022.1002519)
Supplement: Supplementary file 1 [file DataSheet_1.zip › Supplementary Material/Supplementary figure legends.docx]

**Figure S1** Bayesian tree of Rubiaceae based on *rbc*L. The numbers on branches indicate posterior probability

**Figure S2** (A) Plot of geographical distance against genetic distance for 23 populations of *H. lichiangensis.* (B) Mismatch distribution of haplotypes based on pairwise sequence difference against the frequency of occurrence for *H. lichiangensis*. The solid line shows the observed values and the dashed line represents expected values under a model of sudden (step wise) population expansion

**Figure S3** The receiver operating characteristic (ROC) curve and the area under ROC curve (AUC) of the model. (A): LIG; (B): LGM; (C): Present; (D): In 2070s
