# Supplementary material for: Phylogeography of Himalrandia lichiangensis from the dry-hot valleys in Southwest China
Source: Front Plant Sci. 2022 Oct 17;13:1002519. doi: 10.3389/fpls.2022.1002519 (PMC9618719; doi:10.3389/fpls.2022.1002519)
Supplement: Supplementary file 1 [file DataSheet_1.zip › Supplementary Material/Supplementary table.docx]

| Location | | | | | cpDNA | | | ITS | | | *CAMX* | | |
| --- | --- | --- | --- | --- | --- | --- | --- | --- | --- | --- | --- | --- | --- |
| Pop code | Lat. (N) | Long. (E) | Alt. (m) | N | Haplotypes (No.) | *P*i×10^3^ | *H*d | No. | *P*i×10^3^ | *H*d | No. | *P*i×10^3^ | *H*d |
| AS | 23.642 | 103.716 | 1508 | 20 | H1 | 0 | 0 | T1 | 0 | 0 | C1 | 0 | 0 |
| CH | 25.662 | 102.651 | 1674 | 20 | H2 | 0 | 0 | T2, T3 | 0.760 | 0.097 | C2 | 0 | 0 |
| DL | 26.686 | 100.213 | 2231 | 20 | H3, H4 | 0.06 | 0.100 | T2, T4 | 0.150 | 0.097 | C2 | 0 | 0 |
| EY | 28.063 | 100.282 | 2228 | 20 | H5, H6 | 0.03 | 0.100 | T2 | 0 | 0 | C2 | 0 | 0 |
| HD | 23.572 | 103.940 | 1556 | 4 | H1 | 0 | 0 | T1 | 0 | 0 | C1 | 0 | 0 |
| HL | 26.890 | 100.237 | 2567 | 20 | H7 | 0 | 0 | T2, T3, T5 | 1.160 | 0.190 | C2 | 0 | 0 |
| HP | 26.107 | 100.275 | 1670 | 20 | H8, H9 | 0.690 | 0.337 | T1, T6, T7, T8 | 3.200 | 0.542 | C2 | 0 | 0 |
| HT | 24.975 | 100.947 | 1537 | 20 | H8, H10, H11 | 0.340 | 0.395 | T9 | 0 | 0 | C3 | 0 | 0 |
| JP | 26.224 | 102.432 | 2059 | 20 | H1, H12, H13 | 0.290 | 0.426 | T2 | 0 | 0 | C4 | 0 | 0 |
| JZ | 26.029 | 100.421 | 1498 | 20 | H14 | 0 | 0 | T10, T11, T12 | 1.310 | 0.349 | C2 | 0 | 0 |
| KD | 25.484 | 102.799 | 1924 | 20 | H2 | 0 | 0 | T2 | 0 | 0 | C1 | 0 | 0 |
| LD | 30.055 | 102.162 | 1395 | 20 | H8 | 0 | 0 | T13 | 0 | 0 | C5 | 0 | 0 |
| LZ | 24.683 | 101.956 | 1203 | 20 | H15 | 0 | 0 | T1, T14 | 0.300 | 0.097 | C2 | 0 | 0 |
| MD | 25.746 | 100.858 | 1693 | 20 | H16 | 0 | 0 | T1, T3, T15, T16 | 1.430 | 0.632 | C6, C7, C8, C9, C10, C11 | 1.990 | 0.696 |
| MJ | 28.225 | 100.252 | 2186 | 20 | H17 | 0 | 0 | T2 | 0 | 0 | C12 | 0 | 0 |
| NL | 28.059 | 100.520 | 1868 | 20 | H8 | 0 | 0 | T13 | 0 | 0 | C12 | 0 | 0 |
| SG | 26.870 | 99.975 | 1797 | 20 | H18 | 0 | 0 | T2 | 0 | 0 | C12 | 0 | 0 |
| SY | 25.881 | 101.120 | 1600 | 20 | H14, H19 | 0.170 | 0.189 | T1, T6, T17, T18 | 3.680 | 0.682 | C2 | 0 | 0 |
| WM | 25.937 | 101.769 | 1284 | 20 | H1 | 0 | 0 | T1, T3, T5, T19, T20, T21 | 5.220 | 0.779 | C5, C13, C14 | 0.440 | 0.190 |
| XG | 24.489 | 101.978 | 1232 | 14 | H15 | 0 | 0 | T1 | 0 | 0 | C2 | 0 | 0 |
| XS | 24.265 | 103.503 | 1650 | 20 | H1 | 0 | 0 | T1 | 0 | 0 | C1 | 0 | 0 |
| XY | 26.908 | 102.812 | 1447 | 5 | H13 | 0 | 0 | T1, T3, T15, T16 | 2.210 | 0.800 | C13 | 0 | 0 |
| YG | 26.0245 | 101.563 | 1627 | 20 | H13 | 0 | 0 | T1, T8, T19 | 1.780 | 0.272 | C5, C13, C15 | 0.420 | 0.344 |
| Total | | | | |  | 1.450 | 0.917 |  | 5.680 | 0.761 |  | 2.860 | 0.754 |

**Table S1** Details of sampling information, the composition of haplotypes, *H*d and *P*i. Notes: cpDNA: Chloroplast DNA; nDNA: Nuclear DNA; *CAMX*: Calmodulin; ITS: Internal Transcribed Spacers; *H*d: haplotype diversity; *P*i: nucleotide diversity; N: number of sampled individuals per population.
