# Supplementary figures and images for: Phylogeography of Himalrandia lichiangensis from the dry-hot valleys in Southwest China
Source: Front Plant Sci. 2022 Oct 17;13:1002519. doi: 10.3389/fpls.2022.1002519 (PMC9618719; doi:10.3389/fpls.2022.1002519)

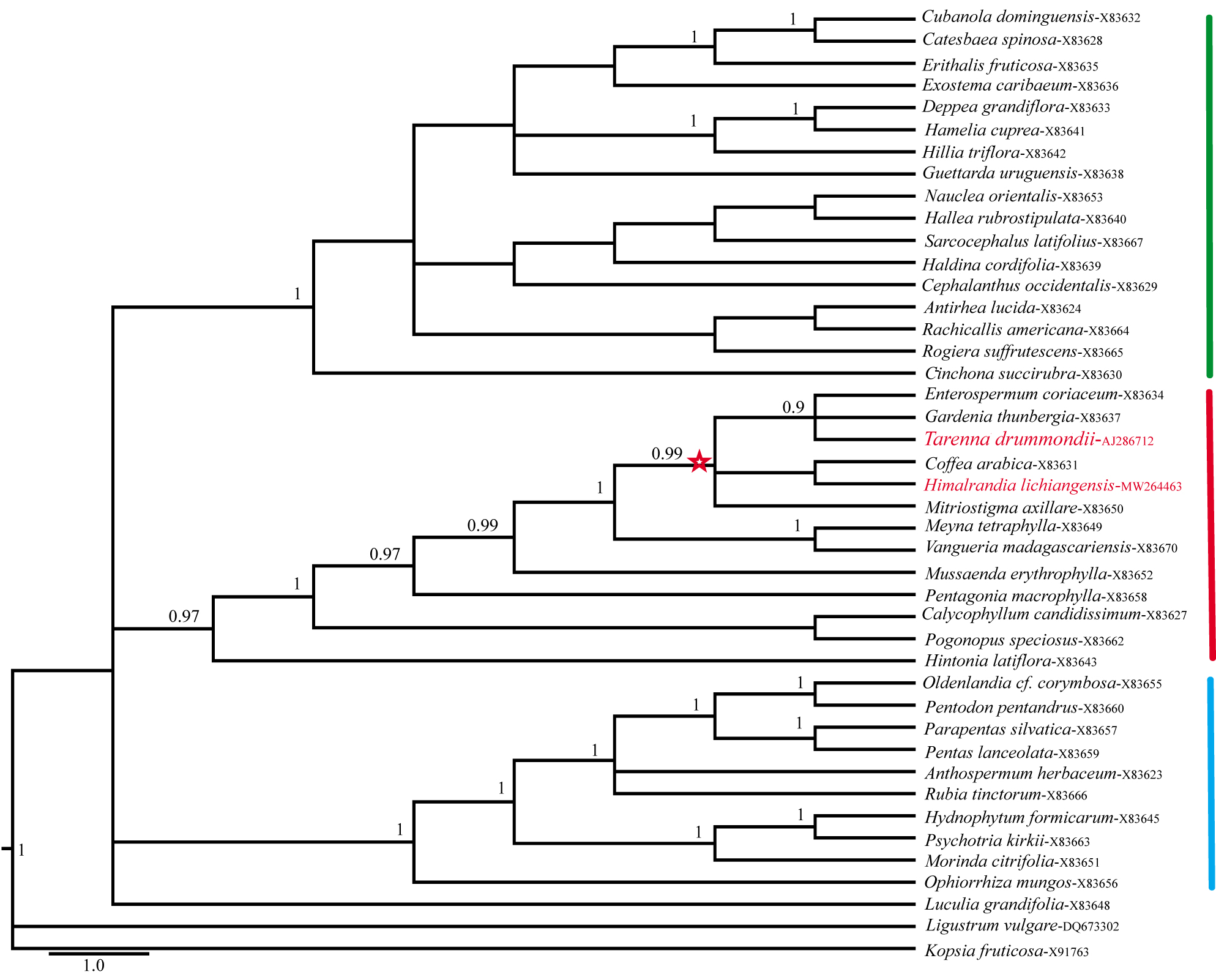

Supplement: Supplementary file 1 [file DataSheet_1.zip › Supplementary Material/Figure S1.jpg]

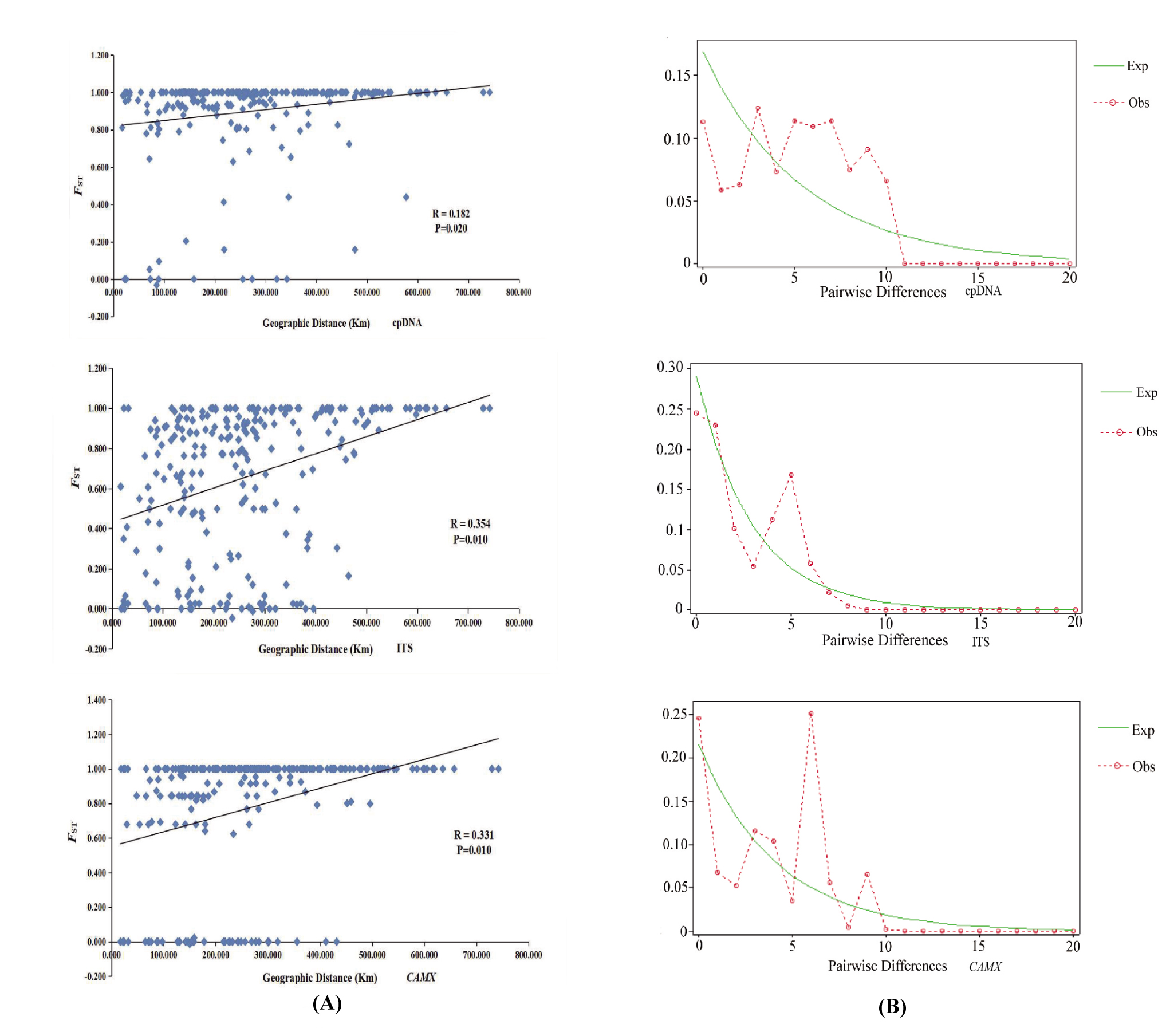

Supplement: Supplementary file 1 [file DataSheet_1.zip › Supplementary Material/Figure S2.jpg]

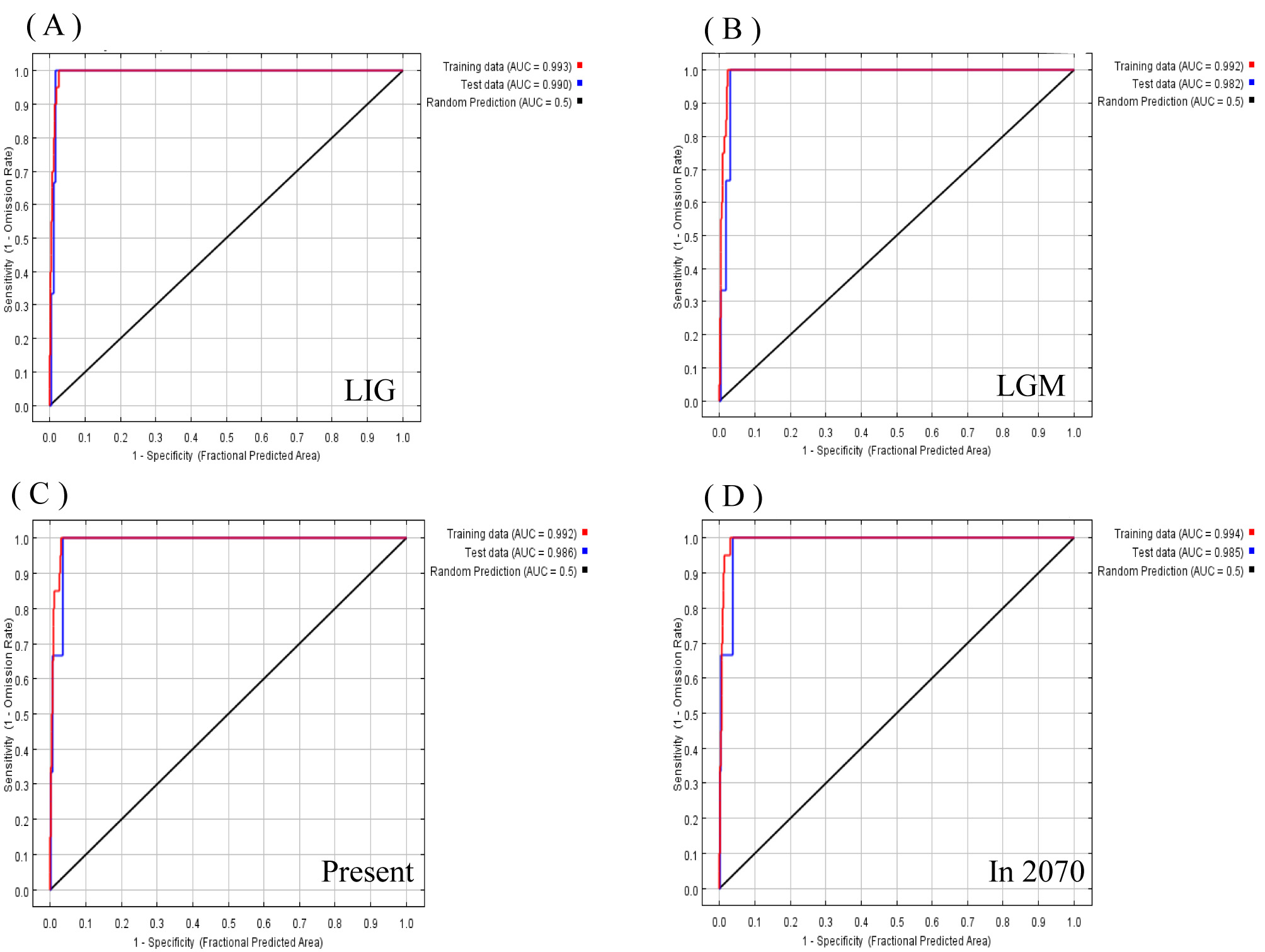

Supplement: Supplementary file 1 [file DataSheet_1.zip › Supplementary Material/Figure S3.jpg]
